# Metropolitan Hospital

**Published:** 1902-04-26

**Authors:** 


### METROPOLITAN HOSPITAL.

### LORD HILLINGDON ACCEPTS A TREASURERSHIP.

Donations from Officers Serving in South Africa.

A large number of those whose subscriptions or whose services—or both—are cheerfully given to the Metropolitan Hospital, Kingsland Road, assembled at the festival dinner of that institution at the Whitehall Rooms of the Hôtel Métropole on Thursday evening, April 10th, and did so to good purpose, for their presence and help put a finishing touch to a list of contributions which reached a total not very far short of £3,000. The chair was occupied by the Right Hon. Lord Hillingdon, who was supported on his right by Mr. C. J. Thomas (chairman of the hospital), others at the head table including Mr. E. Flower, M.P., Sir Edmund Hay Currie, Mr. J. R. Pike, Mr. C. Fitch Kemp, J.P., Mr. H. Wagner, Mr. A. E. Hoare, Mr. H. Gardner, Mr. F. S. Franklin, Mr. A. Lister Harrison, J.P., Mr. D. C. Defries, Mr. R. T. Wragg, Mr. J. Harrison Dakin, Mr. Aylett Moore, Mr. C. Cotes, Mr. J. Lowles, Mr. H. Lovegrove, Mr. T. M. Merriman, Mr. R. M. Cunningham, Mr. F. W. Fry, Mr. V. S. Woods, Col. J. Pelly Fry, and Col. J. S. Young. At the opposite ends of the several tables were Mr. F. Snowden, Mr. D. H. Goodsall, Rev. E. A. B. Sanders, Dr. A. Haig, and Mr. C. H. Byers (secretary and house governor).

After the customary loyal toasts,

The Chairman proposed "Prosperity to the Hospital." Referring to his early recollections in connection with the institution, he mentioned, amid applause, the name of Joseph Fry, to whom the hospital was more indebted than he could say. Mr. Joseph Fry was continually in consultation with his father at his place of business, and to the exertions he made on behalf of the hospital was largely due the happy position in which it stood. The present situation of the hospital was somewhat removed from the ken of most of those whom he was now addressing. "It is a long way out there," observed his lordship, raising a smile, "in the direction from which the wind generally blows at this time of the year—the north-east." Anyone who went there—and he had recently had the privilege of going over the hospital—would recognise at once the enormous want that the institution fulfilled in a very poor and very rising district. With the exception of the German Hospital, which was naturally devoted more to special patients, there was no hospital other than the Metropolitan within nearly two miles. No hospital could be better situated and certainly none could be more airy, better ventilated, more cheerful or better equipped. The amount of good the hospital had done could hardly be reckoned by mere figures, but they might give a little idea in this direction. When the hospital was instituted there were only 12 beds; 247 in-patients were treated, while the attendance of out-patients was 43,000. At the end of last year, 1901, there were 111 beds, there having been 1,131 in-patients and 111,962 attendances of out-patients. (Applause.) They were apt to gauge the work of a hospital by the number of beds it maintained, but that was not its only work. There were hundreds and thousands of people who were not sufficiently ill to be treated in a hospital, whose means and the hardness of whose lives did not permit them to lie up entirely, but who could get strength, comfort, consolation, and sufficient recuperation to go on with their work by attending at an hospital from time to time, and in that respect 111,000 people had been benefited by the hospital. The committee were able to open additional beds last year, mainly due to the fact that the trustees of the Zunz bequest had most generously promised to give £10,000 in yearly instalments of £1,000 each, in order to enable the committee to open the Annie Zunz Ward. (Applause.) This was no mean testimony to the hospital. There was a Jewish ward where Jewish patients were treated with strict observance of their religion, and this was an important feature in that part of London where the hospital was situated. The convalescent home—delightfully situated in Kent—given a few years ago by Mr. Passmore Edwards, and erected upon land most generously presented by Mr. Cornwallis, contained 18 beds and had done excellent work. The Ladies' Association, of which the Duchess of Argyll was president, and which was founded in connection with the hospital in 1899, now numbered 150 members. It had proved of the greatest possible assistance to the hospital, having given £500 to the general fund and £200 to the convalescent home. The Prince of Wales's Fund voted £481 to the hospital in 1897, and in 1901 £1,000 was received from this source as a donation and £500 as a subscription. (Applause.) In further remarks the Chairman expressed regret at the absence of Mr. Leopold de Rothschild, one of the treasurers, who, he remarked, had always had the welfare of the hospital thoroughly at heart. Proceeding, he said those who went through the hospital could not but be struck by the imperative necessity of having a nurses' house apart from the hospital. If this could be established they would be able to open a new ward. A ward was at present relegated to the nurses for the simple reason that there was no house for them to live in. His Lordship pointed out that, for the purpose of establishing such a house, they had not to buy land at a high price—the land was already there, and belonged to the hospital; all that was needed was the erection of a house, and he did not think it was too much to ask those present to create a fund for that purpose. At present it was impossible for the nurses to do their work so well as they would be able to if a proper house were provided. It was only right that they should be taken a little way from their daily labours and the constant drudgery of the sick bed. They would come back feeling fresher, and the patients would derive benefit just as much as the nurses themselves. If this house were provided the hospital would be as complete in its way as any hospital in London. With the toast his lordship coupled the health of the Management Committee.

Mr. C. J. Thomas, C.C., chairman of the hospital, who was cordially received, expressed very great gratification at seeing Lord Hillingdon in the chair. It carried them back to the time when his lordship's father was treasurer of the institution. (Loud applause.) It was many years ago, in the early part of the late Lord Hillingdon's treasurership, that they moved into the present building, and he remembered the very great interest which that gentleman took in the responsible work of extending the operations of the hospital in its present situation. Many things had to be considered, while the outlay necessitated was very large, and his lordship's wise counsel and experience were a very great advantage. It would, he was sure, be a matter of much satisfaction to those present to know that the chairman of the present dinner had consented to be in future one of the joint treasurers of the institution. (Loud applause.) Amplifying what the Chairman had said as to the work done by the hospital, Mr. Thomas mentioned that the provident department, instituted some fifteen years ago at the suggestion of their kind friend Sir Hay Currie—(applause)—had done admirable work which was increasing from year to year. Indeed this work had increased so much that it had become necessary to make special provision for the department, and this they were making in the erection of suitable premises contiguous to the present hospital building. The provident patients had the advantage of being, if necessary, attended in their own homes, and in this connection 5,700 home visits had been paid by the medical officers of the department during the past year. The work of the institution had increased to such an extent as to become almost an embarrassment to the committee, and they had therefore been much gratified in the past year at the liberal donations made by the trustees of the Zunz bequest. This had enabled them to open the Annie Zunz ward for twelve in-patients, and with the assistance given by the Prince of Wales's Fund they had opened an additional ten beds. It was very satisfactory that when they undertook larger responsibilities people were found ready to come forward and support them. They did not go into the North-east of London because it was a neighbourhood which was able to contribute to their funds, but because it was a part of the great City where it was most necessary for hospital accommodation to be provided. (Applause.) Increased contributions did not come in immediately they extended their accommodation; indeed for some years they were gradually getting more deeply into debt. Their former respected chairman, Mr. Joseph Fry, however, worked very hard and interested his friends to such an extent that, although they did not see the happy and satisfactory result during his lifetime, yet it began to come to them very soon afterwards, and the grave anxiety which those connected with the hospital experienced some few years ago had since been very much relieved by most generous contributions.—(Cheers.)—In further remarks the speaker mentioned the excellent work of the Ladies' Association and the Committee of Management, appealed for increased annual subscriptions, and generally commended the work of the hospital to the increased sympathy of those present. Before sitting down he proposed the health of the chairman, which was accorded a "bumper," including musical honours.

Responding, the Chairman announced, to the evident gratification of all his hearers, that he had undertaken the object of establishing the nurses' home, and that it would be his endeavour to carry the project through without in any way interfering with the general work and finances of the hospital. He added: "If I worry you all to death in trying to accomplish this you must accept that as an earnest of the efforts I am making for what I believe to be a thoroughly good cause." (Cheers.)

The Secretary then announced the financial result of the festival, stating that Lord Hillingdon's list, headed by a donation from his lordship of £120, amounted to £1,332; that of Mr. F. Franklin, representing the contributions of 146 people, came to £1,169; and that of Mr. D. C. Defries to £142 18s., the grand total being £2,806. This total included £20 which had come to hand from across the sea in a way that the following letter, read by the secretary, will explain. It was written by an officer serving with the 32nd Company of the Imperial Yeomanry, Col. Byng's Column, Field Force, S.A., the said officer being a member of the committee of the hospital:—"A I happened a few days ago to be handed a tenner to give to any charity I liked by an officer on this column, I at once bethought myself of you, and am adding another one myself. So now have very much pleasure in handing you a cheque for £20. Wishing you a very prosperous new year."

The other toasts were, "The Hospital Staff" (in proposing which the Rev. E. A. B. Sanders promised, on behalf of the Ladies' Association, a contribution of £100 towards Lord Hillingdon's scheme for establishing a nurses' home) and "The Visitors."

The proceedings were much enlivened with music, Karl Kaps' band giving selections during dinner and the speeches being interspersed with some capitally rendered vocal items.

